# Cytokine profile in *Leishmania*-positive blood donors

**DOI:** 10.1371/journal.pone.0238933

**Published:** 2020-09-23

**Authors:** Adriana de Oliveira França, Luana Silva Soares, Mauricio Antonio Pompilio, Inês Aparecida Tozetti, Camila Mareti Bonin, Maria Elizabeth Moraes Cavalheiros Dorval

**Affiliations:** 1 Laboratory of Clinical Parasitology, Graduate Program in Infectious and Parasitic Diseases, Universidade Federal de Mato Grosso do Sul, Campo Grande, MS, Brazil; 2 Laboratory of Immunology, Universidade Católica Dom Bosco, Campo Grande, MS, Brazil; 3 Hélio Mandetta School of Medicine, Universidade Federal de Mato Grosso do Sul, Campo Grande, MS, Brazil; 4 Laboratory of Immunobiology, Institute of Biosciences, Universidade Federal de Mato Grosso do Sul, Campo Grande, MS, Brazil; Academic Medical Centre, NETHERLANDS

## Abstract

Serum levels of interleukin 2 (IL-2), interleukin 4 (IL-4), interleukin 6 (IL-6), interleukin 10 (IL-10), interleukin 17 (IL-17), interferon gamma (IFN-γ), tumor necrosis factor α (TNF-α), and interleukin 1β (IL-1β), cytokines involved in the immune response, were investigated in 75 *Leishmania*-positive blood donors living in endemic areas. Based on their status in 2011 and 2015, the subjects were clustered into three groups: positive for at least one diagnostic method in both years, but lacking clinical progression to disease (G1); positive on at least one method in 2011 but negative in 2015 (G2); negative on all methods in both years (G3). Donors were interviewed for sociodemographic data collection and underwent clinical evaluation and laboratory tests. Serum cytokines were quantified using a CBA Flex set (BD Biosciences). Significant differences were found for all the cytokines evaluated, with lower concentrations in consistently *Leishmania*-negative individuals. The exception was IFN-γ, with similar levels among all donors. No changes consistent with active disease were observed in the laboratory results for *Leishmania*-positive donors who underwent clinical evaluation, none of whom progressed to disease. This suggests that infection control is associated with serum IL-17 levels. Resolution of *Leishmania* infection in positive donors may be related to high levels of IL-17 and low levels of IL-10, highlighting the role played by IL-17 in asymptomatic *Leishmania*-infected individuals.

## Introduction

Visceral leishmaniasis (VL) is a serious public health problem in Brazil. The disease can have asymptomatic, subclinical, or oligosymptomatic presentations. The acute form has classic manifestations, including intermittent fever, weight loss, hepatosplenomegaly, anemia, leucopenia, and hypergammaglobulinemia [1].

Up to 85% of individuals can control infection spontaneously; remaining asymptomatic or developing the oligosymptomatic form that eventually evolves with self-resolution [2].

Clinical forms reflect the relationship between parasite multiplication in macrophages and host immune response [3]. The mechanisms determining the progression of infection to disease have not been fully elucidated, but cellular immune response, seems play a crucial role [4, 5].

Evaluation of serum cytokine production has been regarded as a promising tool to distinguish clinical forms of VL, given the positive relationship with disease progression [6].

In the present cross-sectional investigation, serum levels of interleukin 2 (IL-2), interleukin 4 (IL-4), interleukin 6 (IL-6), interleukin 10 (IL-10), interleukin 17 (IL-17), interferon gamma (IFN-γ), tumor necrosis factor α (TNF-α), and interleukin 1β (IL-1β), cytokines, were investigated in *Leishmania*-positive blood donors living in endemic areas.

## Materials and methods

### Study design and subjects

The investigation was conducted at the José Scaff Hematology and Hemotherapy Center of Mato Grosso do Sul (Hemosul), in Campo Grande, Mato Grosso do Sul state, Midwest Brazil. The study included donors considered fit, in clinical and laboratory terms, for blood donation—i.e., negative results in all screening tests and no signs, symptoms, or history of leishmaniasis.

From a previous study conducted in 2011, samples from 430 donors were selected and tested for *Leishmania* using the indirect fluorescent antibody test (IFAT; Biomanguinhos, Rio de Janeiro), enzyme-linked immunosorbent assay (rK39 ELISA), rK39 rapid immunochromatographic test (ICT; Kalazar-Detect), and polymerase chain reaction. *Leishmania* infection was defined as positivity on at least one of these tests [7].

Of 178 positive donors, 50 who had at least one positive test for *Leishmania* sp., plus 25 donors who were negative on all tests (controls), were selected. In 2015, all 75 donors were invited to visit the Hospital Universitário Maria Aparecida Pedrossian, the teaching hospital of the Universidade Federal de Mato Grosso do Sul, for clinical evaluation, laboratory tests, and fresh blood sample collection, for investigation after four years.

### Clinical data and laboratory tests

The clinical approach was performed by an infectious disease physician at a hospital setting during consultation, through semi-structured interviews to obtain sociodemographic data, pathology history (previous comorbidities), treatment history for VL. Donors called for the second evaluation underwent general and specific physical examination.

Donors suspected of having the disease were examined clinically for investigation and identification of signs and symptoms such as pale mucous membranes, lymph node enlargement, hepatomegaly, splenomegaly, fatigue, weight loss, fever for more than 15 days, increase abdominal volume, and spontaneous bleeding. Laboratory tests for blood count, dosage of total proteins and fractions, alanine aminotransferase (ALT), and aspartate aminotransferase (AST) were performed.

In compliance with protocols of the hospital’s clinical analysis laboratory, blood cells were counted on a Sysmex XE-2100D analyzer and differential white cell counts were confirmed in blood smears stained with May–Grünwald–Giemsa. Anemia was defined as hemoglobin level ≤13.5 g/dL for males and ≤12 g/dL for females. Leukopenia was defined as cell counts ≤4500/mm^3^ and thrombocytopenia as ≤150 000/mm^3^. Upper limits for AST and ALT were 40 and 41 U/L, respectively, as per the enzymatic method employed. In the colorimetric method adopted, reference ranges for total proteins and albumin were 6–8 and 3.5–5.5 g/dL, respectively.

### Cytokine quantification

Whole blood samples were collected, sera were separated and stored at –80°C for quantification of IL-2, IL-4, IL-6, IL-10, IL-17, IFN-γ, TNF-α, and IL-1β.

The cytokines were quantified by flow cytometry (FACSCanto II system, BD Biosciences, San Jose, CA, USA) using a BD Cytometric Bead Array Human Th1/Th2/Th17 cytokine kit (CBA, BD Biosciences) according to manufacturer's instructions. FCAP Array, v. 3.0, software (Becton Dickinson, Franklin Lakes, NJ, USA) was used for data analysis. Results were expressed as pg/mL, based on standard concentration curves.

Cytokine levels were quantified in serum samples obtained in 2011 and 2015 from 75 blood donors shown to be *Leishmania-*positive or negative (control group) in serological and molecular tests.

Samples were categorized into three groups, based on donor status, as follows. Group 1 (G1) included donors who tested positive for *Leishmania* on at least one serological or molecular method in 2011 and positive on the same methods in 2015, but who did not evolve to clinical disease. Group 2 (G2) comprised donors who tested positive on at least one serological or molecular method in 2011, but negative on the same methods in 2015. Group 3 (G3) included donors negative for *Leishmania* on all methods in both years.

### Statistical analysis

The Chi-squared test, supplemented with Yates correction or Fisher exact test, was performed to identify possible associations between variables. Prevalence ratios (PR) were calculated with respective confidence intervals (CI) of 5%.

Cytokine quantification data were analyzed using the GraphPad Prism 6.0 software (San Diego, CA, USA). Differences were considered statistically significant when *p* < 0.05. Given the non-parametric nature of the data set, Wilcoxon and Mann–Whitney tests were performed to compare groups.

For multiple comparisons, differences between the groups were calculated according to the Kruskal-Wallis test.

### Ethical considerations

The study was approved by the UFMS Research Ethics Committee (permit 1976, CAAE 0037.0.049.049–11). All subjects voluntarily signed a statement of informed consent for the collection of data and received their exam results, along with clarifications on clinical and epidemiological features of *Leishmania* infection.

## Results

Ages ranged from 23 to 61 years, with a mean of 36 ± 10.4 years (SD). Males predominated (60.0%). Up until 2015, the subjects had donated blood for a mean period of 4.9 ± 6.2 years (SD), and four (5.3%) had a history of blood transfusion.

Except for one suspected case in 1999, none of the participants had previously been diagnosed with leishmaniasis. Only one donor had fever for more than 15 days. Clinical manifestations were described in Table 1.

**Table 1:** Distribution of blood donors, by sex, age, and clinical manifestations associated with *Leishmania* infection. Campo Grande, MS, Brazil, 2015 (*n* = 73).

Variable	G1(25)		G2(25)		G3(23)		*p*
	n	%	n	%	n	%	
Age (median)*	35	–	35	–	39	–	0,8391
(1st-3rd quartile)	(29–45)	–	(29.5–47.5)		(29–47)	–	
Sex							
Male	15	60,0	17	68,0	11	47,8	0,3619
Female	10	40,0	8	32,0	12	52,2	
Clinical manifestations**							
Fatigue	7	28,0	2	8,0	4	17,4	0,1809
Pallor	3	12,0	4	16,0	0	0	0,1502
Weight loss	3	12,0	2	8,0	2	8,7	0,8774
Palpable lymph nodes	3	12,0	1	4,0	0	0	0,1745
Increased abdominal volume	1	4,0	1	4,0	3	13,0	0,3643
Prolonged fever (≥15 days)	1	4,0	0	0	0	0	0,3778

*Chi-squared test. Differences between groups were calculated using the Kruskal–Wallis test for multiple comparisons.

**Values of *n* express the number of donors exhibiting clinical manifestations. Two subjects who tested negative in 2011 but positive in 2015 were excluded.

Clinical and laboratory findings ruled out active VL at the time of the evaluations. All participants were in good health, with no signs of changes consistent with the disease.

**Fig 1:**
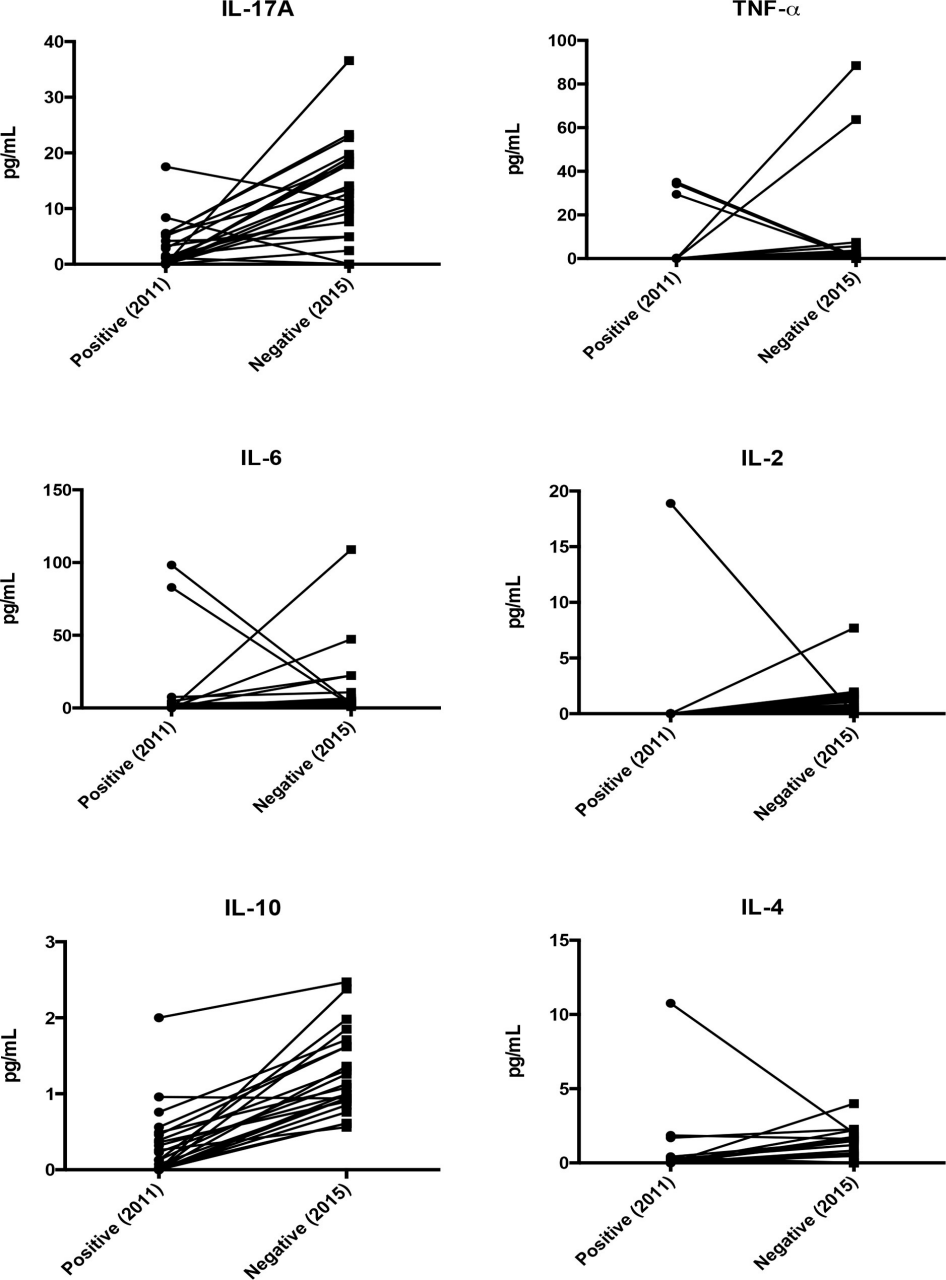
Serum cytokine levels in blood donors positive for *Leishmania* in 2011 and negative in 2015.

In Table 2, serum cytokine levels are grouped by maintenance or change of *Leishmania* infection status in 2011 and 2015. Significant increases in TNF-α, IL-10, IL-6, and IL-2, were observed in G1 on the second evaluation. In G2 IL-2, IL-17, IL-6, IL-10, and IL-4 levels increased significantly in 2015 (Table 2 and Fig 1). In G3, IL-1β concentrations decreased significantly (Table 2).

**Table 2:** Distribution of serum cytokine levels, by maintenance or change of *Leishmania* infection status in a four-year period. Campo Grande, MS, Brazil, 2011–2015 (*n* = 73).

Cytokine	Mean ± SD (2011)	Mean ± SD (2015)	p*
**G1 (*n* = 25): 2011 (+) → 2015 (+)**			
IL-17A	5.10 ± 7.92	6.24 ± 9.14	0.6436
IFN-γ	0.12 ± 0.47	0.12 ± 0.32	>0.9999
TNF-α	0.43 ± 1.96	16.10 ± 34.17	0.0038
IL-10	0.40 ± 0.51	1.31 ± 1.02	0.0004
IL-6	0.71 ± 1.22	16.27 ± 39.13	<0.0001
IL-4	0.35 ± 1.13	0.43 ± 0.78	0.8203
IL-2	0.33 ± 1.54	0.82 ± 0.94	<0.0007
IL-1β	3.91 ± 14.32	2.14 ± 3.91	0.4801
**G2 (*n* = 25): 2011 (+) → 2015 (–)**			
IL-17A	2.53 ± 3.97	12.26 ± 8.73	<0.0001
IFN-γ	0.05 ± 0.21	0.12 ± 0.26	0.2266
TNF-α	3.96 ± 10.94	7.35 ± 21.08	0.1439
IL-10	0.29 ± 0.45	1.25 ± 0.52	<0.0001
IL-6	8.13 ± 24.98	10.89 ± 22.81	0.0011
IL-4	0.63 ± 2.17	1.02 ± 0.99	0.0019
IL-2	0.76 ± 3.78	1.20 ± 1.50	0.0001
IL-1β	12.41 ± 30.58	4.80 ± 8.69	0.4304
**G3 (*n* = 23): 2011 (–) → 2015 (–)**			
IL-17A	5.36 ± 4.27	6.70 ± 8.16	0.9429
IFN-γ	0.09 ± 0.16	0.04 ± 0.12	0.3613
TNF-α	0.36 ± 1.18	0.05 ± 0.22	0.1250
IL-10	0.63 ± 0.48	0.63 ± 0.53	0.6924
IL-6	2.33 ± 6.26	1.14 ± 1.15	0.4291
IL-4	0.46 ± 0.42	0.36 ± 0.52	0.3060
IL-2	0.10 ± 0.18	0.21 ± 0.34	0.1616
IL-1β	1.80 ± 5.28	0.70 ± 2.20	0.0015

*Differences were considered statistically significant at *p* < 0.05 (Wilcoxon’s test).

G1: *Leishmania*-positive in both years. G2: positive in 2011, negative in 2015. G3: negative in both years. Two subjects who tested negative in 2011 but positive in 2015 were excluded.

In G2 and G3, significant differences were found for all the cytokines evaluated, with lower concentrations in *Leishmania*-negative individuals, with exception to IFN-γ (Table 3).

**Table 3:** Serum cytokine levels among blood donors with negative *Leishmania* status on the second evaluation. Campo Grande, MS, Brazil, 2015 (*n* = 48).

Cytokine	Mean ± SD		*p*
	G2: 2015 (–)	G3: 2015 (–)	
IL-17A	12.26 ± 8.73	6.70 ± 8.16	0.0134
IFN-γ	0.12 ± 0.26	0.04 ± 0.12	0.227
TNF-α	7.35 ± 21.08	0.05 ± 0.22	<0.0001
IL-10	1.25 ± 0.52	0.63 ± 0.53	<0.0001
IL-6	10.89 ± 22.81	1.14 ± 1.15	<0.0001
IL-4	1.02 ± 0.99	0.36 ± 0.52	0.0079
IL-2	1.20 ± 1.50	0.21 ± 0.34	<0.0001
IL-1β	4.80 ± 8.69	0.70 ± 2.20	0.0004

Differences were considered statistically significant at *p* < 0.05 (Mann—Whitney test).

G2: *Leishmania-*positive in 2011, negative in 2015. G3: negative in both years.

## Discussion

The mechanisms by which asymptomatic individuals can contain parasite multiplication and control infection remain unknown [3, 8]. In terms of parasite control, only the joint effect of cytokines, but not their isolated effects, could be evaluated in the present study, but the results suggest that infection control may involve a balance between IL-17 and IL-10.

The role of IL-17 in leishmaniasis remains controversial. While its association has been reported as conferring protection against *L*. *donovani* in humans [9, 10], has also been shown to contribute in the pathogenesis of VL by the same agent [11].

Infection control and disease progression have been associated with Th1 and Th2 responses. However, depending on the *Leishmania* species involved and the genetic history of host susceptibility or resistance, Th17 cell responses can also play a role [12].

In the present study, none of the subjects who were *Leishmania*-positive on both evaluations and had no changes in cytokine levels compatible with active leishmaniasis progressed to the disease. This finding is in agreement with Pitta *et al*. [10], who found a higher proportion of resistant individuals among patients exhibiting increased IL-17 levels, and a 20-fold higher risk of VL development in those with low concentrations.

Because patients with VL submitted to treatment develop a strong Th1 response, they exhibit decreased levels of IL-17, even if higher than in healthy individuals [9], and re-exposure to the parasite confers them protection [13]. Individuals who recover from leishmaniasis may therefore acquire lasting protection against reinfection, a benefit that may correlate with strong IL-17 production [14].

Protective immune response can be achieved through the production of pro-inflammatory cytokines associated with the Th1 profile, which prompt infected macrophages to eliminate parasites by releasing nitric oxide and free radicals [3]. IFN-γ can also act with IL-17 to potentiate this effect on macrophages, in addition to being associated with regulatory T-cell inhibition. In mice experimentally infected with *L*. *infantum*, infection control in the absence of IL-10 was found to be regulated by IL-17 [9].

Gama *et al*. [15] reported high levels of IFN-γ in oligosymptomatic patients who evolved to spontaneous cure, as well as in individuals with no signs of disease severity who had higher levels of IL-10 too. The low levels of IFN-γ found in our samples may be explained by insufficient stimulus for production of this cytokine, given that parasite burden in carriers is lower than in patients with active disease [9].

TNF-α release depends on the generation of cytokines such as IL-4 and IFN-γ [16], was detected at low concentrations in the present samples. Interpreting these findings can be a daunting task, since high TNF-α levels have been reported for Brazilian patients with active VL [6], while Indian counterparts exhibited low levels of this cytokine [17].

Gama *et al*. [4] found that higher TNF-α levels during active disease contribute to discrete clinical manifestations in oligosymptomatic patients. In the present samples, the TNF-α concentrations in G1 and G2 may have been associated with the concomitant increases detected in IL-10, which plays a regulatory role.

Comparing cured and asymptomatic subjects, Costa *et al*. [6] found no differences in serum IFN-γ or TNF-α levels, which is in agreement with studies conducted in Iran [18] and Brazil [3]. TNF-α [19] and IFN-γ levels [17, 18] have been reported as reliable markers of cured VL. Cure is followed by the establishment of protective mechanisms similar to those found in asymptomatic individuals [3].

The absence of disease in asymptomatic individuals might be explained by an intrinsic ability to achieve a balance between immunoregulatory profile (IL-10 and IFN-γ), leading to parasite elimination with no tissue damage [20].

In our asymptomatic subjects, low levels of IFN-γ and IL-10 were detected, corroborating findings by Peruhype-Magalhães *et al*. [21]. Low concentrations of IL-10 and IFN-γ reflect an intermediate status between disease and protective immunity, indicating the important role of these cytokines in controlling parasite growth [18].

Individuals infected with *Leishmania* may exhibit weaker immune responses, owing to decreased IL-2 and IFN-γ production [22], with consequent inefficient parasite elimination. In the present study, IL-2 was assumed to influence, even at low serum levels, the self-limited evolution of clinical manifestations, culminating in the spontaneous resolution of infection in asymptomatic individuals [2, 23]—a role shared by IL-17, as previously discussed.

These results are in agreement with Belosevic *et al*. [24] in that IL-2 and IFN-γ, even at basal levels, may promote phagocytic mechanisms in macrophages. On the other hand, some patients with undetectable IL-2 and IFN-γ levels and low IL-10 concentrations have exhibited signs of disease severity and died [15].

Using soluble *Leishmania* antigen to stimulate whole blood samples from asymptomatic donors, Ibarra-Meneses *et al*. [23] found IFN-γ and IL-2 concentrations to increase significantly, compared with negative controls. This finding led the investigators to posit IL-2 as a novel marker for detecting asymptomatic individuals. Our results, however, do not corroborate this claim, since neither IL-2 nor IFN-γ concentrations proved high, and thus failed to serve as epidemiological markers for asymptomatic individuals living in an endemic area.

Examining peripheral blood mononuclear cells, Pitta *et al*. [10] demonstrated that in healthy individuals exposed to *L*. *donovani* the levels of IL-6 (*p* < 0.001) and IL-1β (*p* < 0.001) in cell cultures were higher in VL-resistant subjects than in those who developed the disease. These findings correlate with our results, which showed significant increases of IL-6 and IL-1β in *Leishmania*-positive subjects who did not develop active disease. The role of IL-6 in maintaining a balance between T-cell responses during disease has been emphasized by Bhattacharya and Ali [25]. Also, this cytokine is possibly associated with suppression of Th2 responses [26].

However, IL-6 has been strongly associated with leishmaniasis severity and deaths, which may be explained by TNF-α inhibition in early infection [26]. In our asymptomatic subjects, however, both IL-6 and TNF-α levels proved high, in contrast with the lower IL-6 concentrations found by Santos *et al*. [27] in severe VL patients who subsequently died.

In the present study, IL-1β levels were higher in subjects who had come into contact with the parasite than in those who remained negative throughout the study period. This finding endorses reports that IL-1β is associated with host resistance, showing that the inflammasome is activated in response to *Leishmania* infection [28]. It also corroborates previous reports of inflammasome activation being critical for the control of *L*. *amazonensis*, *L*. *braziliensis*, and *L*. *infantum chagasi* replication *in vivo* [29].

Because IL-4 plays a crucial role in VL susceptibility and progression [30] and is involved in the regulation of IFN-γ production, its absence has a positive effect on the progression from subclinical disease to spontaneously resolved infection [8]. In our study period, IL-4 levels increased in previously infected individuals. Among our subjects, however, no significant differences in this cytokine were observed, suggesting that IL-4 does not have discriminative expression during infection evolution.

A major challenge observed in *Leishmania*-positive individuals is the lack of a single predictive factor valid for both oligosymptomatic and asymptomatic forms. Production of susceptibility and resistance cytokines therefore plays a key role in the outcome of infection in these individuals, whose clinical features contrast with those of patients with clinically manifest VL [4, 15].

The combined action of IL-4 and IL-10, both at high concentrations, may potentiate their effects on the inhibition of the leishmanicidal activity of macrophages, facilitating parasite multiplication and disease development [3, 31]. This suggests that these levels were not sufficiently high to interfere with the development of an effective immune response, which in turn involves interaction of a more complex network of cytokines in humans than that observed in a murine model, in which Th1 and Th2 are differentiated.

IL-10 has been implicated in suppressing host immunity in human VL, based on the elevated levels of IL-10 seen in plasma and lesion tissue and the role of this cytokine in preventing clearance of *Leishmania donovani* in murine VL models [31, 32].

It remains unclear why 90% of individuals infected with *L*. *infantum* fail to develop the disease, although the efficiency of innate and adaptive cellular immune responses, associated with intrinsic factors in the host, is already known to promote resistance against infection [20, 27].

The fact that IL-17, detected at high levels in our subjects, can generate sufficient inflammatory response to contain the parasite burden, with a positive effect on host resistance [9], might explain the successful control of infection and disease progression observed.

Cytokine profiles of *Leishmania*-positive donors differed from those of consistently negative subjects, in contrast with findings by Hailu *et al*. [33], who found no differences between individuals with asymptomatic infection and healthy subjects.

The complexity of immune responses compounds the challenge of diagnosing *Leishmania* infection, precluding inferences on the mechanisms of reinfection in endemic areas. Lack of knowledge about time of infection and time elapsed between diagnosis and detection of immune responses warrants further investigation to allow deeper interpretation of the present results.

Also, studies in other endemic areas, conducted under the same methodological conditions, should yield gains both in terms of reproducibility and detection of cases of asymptomatic VL. These investigations can also lead to the identification of markers for immunotherapeutic and immunoprophylactic approaches, helping to monitor these individuals and reduce the morbidity and mortality of human VL.

## Supporting information

S1 Data Citokyne level. (XLSX)

S1 File Checklist. (DOC)
